# Retraction Note: p62 improves AD-like pathology by increasing autophagy

**DOI:** 10.1038/s41380-020-0854-x

**Published:** 2021-07

**Authors:** A. Caccamo, E. Ferreira, C. Branca, S. Oddo

The Editor-in-Chief and publisher have retracted this article [1] after an investigation by Arizona State University concluded that Figs. 1c and 4a had been manipulated and falsified by one or more of the authors. The Editor-in-Chief therefore no longer has confidence in the validity of the results and conclusions presented in this article.

A. Caccamo, C. Branca and S Oddo agree with this retraction. E. Ferreira has not responded to correspondence related to this retraction.
